# PCNA Loaders and Unloaders—One Ring That Rules Them All

**DOI:** 10.3390/genes12111812

**Published:** 2021-11-18

**Authors:** Matan Arbel, Karan Choudhary, Ofri Tfilin, Martin Kupiec

**Affiliations:** The Shmunis School of Biomedicine and Cancer Research, The George S. Wise Faculty of Life Sciences, Tel Aviv University, Tel Aviv 69978, Israel; matanarble@gmail.com (M.A.); karan.59832@gmail.com (K.C.); ofritfilin@gmail.com (O.T.)

**Keywords:** RFC, Elg1, Ctf18, PCNA, clamp loading and unloading, ubiquitin, SUMO

## Abstract

During each cell duplication, the entirety of the genomic DNA in every cell must be accurately and quickly copied. Given the short time available for the chore, the requirement of many proteins, and the daunting amount of DNA present, DNA replication poses a serious challenge to the cell. A high level of coordination between polymerases and other DNA and chromatin-interacting proteins is vital to complete this task. One of the most important proteins for maintaining such coordination is PCNA. PCNA is a multitasking protein that forms a homotrimeric ring that encircles the DNA. It serves as a processivity factor for DNA polymerases and acts as a landing platform for different proteins interacting with DNA and chromatin. Therefore, PCNA is a signaling hub that influences the rate and accuracy of DNA replication, regulates DNA damage repair, controls chromatin formation during the replication, and the proper segregation of the sister chromatids. With so many essential roles, PCNA recruitment and turnover on the chromatin is of utmost importance. Three different, conserved protein complexes are in charge of loading/unloading PCNA onto DNA. Replication factor C (RFC) is the canonical complex in charge of loading PCNA during the S-phase. The Ctf18 and Elg1 (ATAD5 in mammalian) proteins form complexes similar to RFC, with particular functions in the cell’s nucleus. Here we summarize our current knowledge about the roles of these important factors in yeast and mammals.

## 1. Introduction

Proper functioning of all cells and organisms requires a stable genome that can withstand internal attacks (such as lesions created by oxidative damage) and external genotoxic insults (chemicals and radiation that may cause DNA lesions). In addition to these challenges, the genome must be accurately copied and properly segregated to the resulting daughter cells during each cell division. Given the size of most genomes, this is a daunting task, as replication must be completed in a reasonable amount of time and involves the dangerous unravelling of the double DNA helix. This situation renders DNA prone to chemical modifications and alterations. Several surveillance and repair mechanisms operate in eukaryotic cells and work together to ensure the stability of the genome [1,2]. When they fail to act appropriately, dire consequences to the cell ensue. Indeed, genomic instability is the hallmark of cancer cells. Most human cancer cells show signs of genome instability, including elevated mutation rates and gross chromosomal rearrangements, including deletions, inversions, and translocations. One of the main proteins in charge of the regulation and coordination of DNA replication and its many complexities is PCNA.

**Figure 1:**
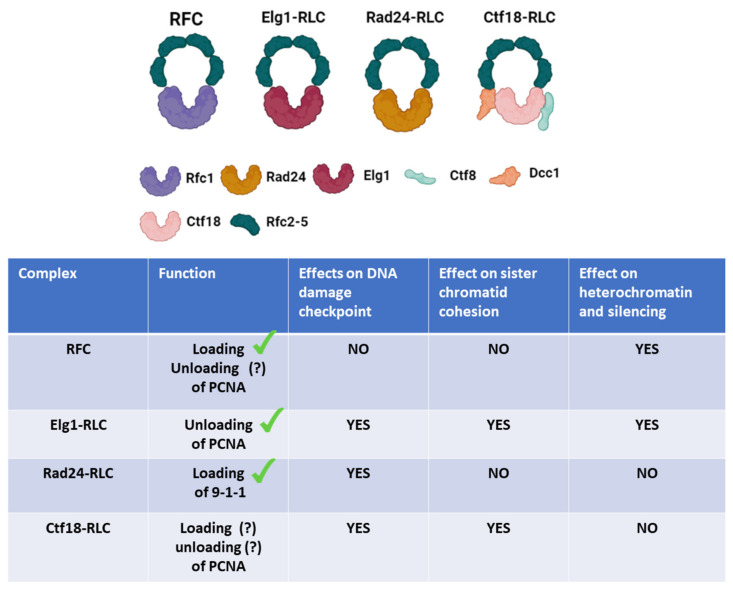
**Schematic representation of RFC and the various RLCs**: the RFC and RFC-like complexes (RLCs) are shown with a table summarizing their known functions and the cellular processes in which they take part.

PCNA (proliferating cell nuclear antigen) is a protein that forms a trimeric ring that encircles the DNA. PCNA plays a significant role in all three major challenges of replication: faithfully replicating DNA even in the presence of DNA damage, preserving and duplicating chromatin structure, and establishing sister chromatid cohesion. Since PCNA functions in so many highly conserved and vital processes, it is crucial to understand its regulation and turnover on the chromatin (previously reviewed in [3,4,5,6]). Three different protein complexes act as clamp loaders/unloaders in eukaryotes. They share four identical subunits (Rfc2-5) and are distinguished by their largest protein, Rfc1, Elg1, or Ctf18. A fourth complex of the same family carries the Rad24 large protein (Rad17 in humans) and plays a role in loading the 9-1-1 PCNA-like checkpoint ring (Figure 1). In this review, we detail what is known about the complex biology of the clamp loaders/unloaders, concentrating on the breakthroughs achieved in the yeast *Saccharomyces cerevisiae*.

## 2. RFC and RFC-like Complexes (RLCs)

As PCNA forms a ring around DNA, it needs to be open to get loaded and unloaded. PCNA loading during DNA replication is carried out by a five-subunit complex, Replication Factor C (RFC). RFC comprises a large subunit, Rfc1, and four small subunits (Rfc2–5) [7]. All the RFC proteins belong to the AAA+ family of proteins and use ATP to exert pressure on the PCNA ring, thus opening it [8]. In vitro, RFC loads PCNA at the junction between ssDNA and dsDNA [7].

Studies in yeast identified, in addition to the canonical RFC complex, three additional RFC-like complexes or RLCs (Figure 1). Two of these are involved in the loading and unloading of PCNA (Ctf18-RLC and Elg1-RLC), whereas the Rad24-RLC (known as Rad17 in mammals) loads the 9-1-1 complex. The 9-1-1 is a PCNA-like complex that is part of the DNA Damage Response. These evolutionarily conserved complexes share the identical four small Rfc2–5 subunits but replace the large Rfc1 subunit with a different protein. Here we summarize what is known about the different clamp loaders/unloaders.

### 2.1. Rfc1

PCNA loading is critical for DNA replication and genomic stability. The timely coordinated loading of PCNA at each origin of replication on the leading strand, and the continuous loading on the lagging strand, are carried out by the RFC (Replication Factor C) complex [9]. All five subunits of RFC (Rfc1–5) are essential for life [10]. They contain a common sequence called the RFC box, including a general Walker-type ATPase motif [11]. Rfc1 contains, besides the RFC box, both N- and C-terminal extensions [12]. The C-terminal region of all the five subunits of the RFC is required to form the complex [13]. The crystal structure and electron microscopy of the RFC complex indicate that the five subunits form an open circular shape with an opening between Rfc1 and Rfc5, making it ideal for interacting with PCNA [14]. The N-terminal region of Rfc1 is unstructured, and its structure and specific function remain mysterious.

The ATP-bound RFC complex binds to and opens the PCNA homotrimeric ring. Unlike many other ATPase proteins, the clamp loader does not use ATP hydrolysis as a force-generation step. Instead, the ATP-bound RFC opens PCNA through binding energy alone [15]. The newly formed intermediate of opened PCNA and RFC binds to gapped or nicked DNA, at which point the ATP is hydrolyzed, freeing the PCNA from RFC and allowing the closure of the PCNA complex to form a circle once again [16,17]. This makes the clamp loader an ATP-dependent protein remodeling switch [18]. Much is still unknown about Rfc1’s activity and function. While its role as the primary loader of PCNA is clear at this stage, there is still conflicting evidence regarding its ability to unload PCNA [19,20,21]. Because Rfc1 is the only major subunit essential for life (*elg1Δ ctf18Δ rad24Δ* mutants, albeit sick, are alive [22]), it stands to reason that it can facilitate itself all the loading and unloading of PCNA when necessary. However, it is also possible that spontaneous disassembly of PCNA from the chromatin is robust enough to allow the cell to survive even without a working PCNA unloader.

By using a method (eSPAN) that measures the enrichment of particular proteins at nascent DNA [23,24], Yu et al. showed that the central location for Rfc1 during replication is at the lagging strand while Ctf18 is mainly located at the leading strand. The bias in Rfc1 location between the leading and lagging strands could be explained by the need of loading PCNA at each Okazaki fragment (see below).

As the RFC complex can probably load and unload PCNA, it is still unclear what regulates its activity. How do cells prevent untimely unloading or loading of PCNA? Moreover, how do they prevent futile cycles of loading and unloading by the RFC complex? Further investigation is crucial to thoroughly understand the regulation and functioning of an RFC complex.

### 2.2. Elg1 (Mammalian ATAD5)

While loading PCNA on the chromatin is pivotal for DNA replication, its unloading is almost of equal importance. The timely unloading of PCNA and the management of PCNA molecules on the chromatin is critical for genomic stability and the expected continuation of the cell cycle [25,26]. The main protein complex in charge of unloading PCNA is the Elg1-RLC, in which Elg1 interacts with the Rfc2–5 subunits instead of Rfc1 [27]. Elg1 (Enhanced Levels of Genomic instability) was identified in several genome-wide screens in yeast that looked for mutants with different phenotypes, all related to genome stability [22,28,29,30,31,32]. Although yeast strains carrying an *ELG1* deletion are alive, they present a wide range of phenotypes, including increased rates of gross chromosomal rearrangements [31] and chromosomes loss [22], increased levels of homologous recombination (HR) events [22,33], and elevated Ty transposition [28]. In addition, deletion of *ELG1* also leads to elongated telomeres [32,34] and sensitizes the cells to various DNA damaging agents [35,36]. These strains also exhibit a high level of spontaneous DNA damage, which can be visualized as high levels of Ddc2 and Rad52 foci [37,38].

Elg1 affects HR in a variety of ways. Deletion of *ELG1* causes high levels of every kind of HR event tested in vegetative cells (no meiotic phenotypes were observed) [22]. Moreover, *elg1Δ* strains are extremely sick in the absence of a functional HR mechanism [22], suggesting that the cells are dependent on HR for survival. However, in the presence of DNA damaging agents, such as MMS, *elg1Δ* mutants show lower induction of HR events than wild type cells [33], and Elg1 is a vital part of the ‘salvage pathway’, an HR mechanism negatively controlled by the Srs2 helicase [39] (see below). Therefore, whereas some HR events are encouraged in the absence of Elg1, others are hampered by its absence. It is still unclear whether different HR sub-pathways are affected differently by the absence of Elg1 or whether the same pathways are differently affected at different times or genomic circumstances.

As with the other RLCs, Elg1 works as a complex together with Rfc2-5 [22,40]. It is worth mentioning that whereas all five RFC subunits (Rfc1–5) are expressed at about the same levels, Elg1 is three-fold less abundant [41]. This is interesting as arguably each PCNA loaded by RFC needs to be unloaded by Elg1-RLC, which implies the need for the same amount of RFC complex as the Elg1-RLC complex. Different protein half-life, catalytic capability, or affinity to the Rfc2–5 complex between Elg1 and Rfc1 may explain the differences. Another possible explanation is that Elg1-RLC is not the only PCNA unloader or that PCNA can unload spontaneously in a robust enough manner. In addition, as *elg1Δ/elg1Δ* diploid mutants show no meiotic phenotypes, it is unclear who is in charge of unloading PCNA during meiotic DNA synthesis.

Does Elg1 work as an ATPase? Proteins of the AAA+ family usually have conserved Walker A and B motifs, which are used for ATP binding and hydrolysis [19,42]. In the yeast Elg1 protein, both motifs deviate from the consensus [43]. The human ortholog of Elg1, ATAD5, differs only in its Walker B sequence. In vitro experiments suggest that binding of ATP and not its hydrolysis is essential for the unloading of the PCNA by ATAD5 and Elg1 [19]. ATAD5-RLC works similarly to the RFC complex [16,17]: the binding and opening of PCNA by the ATAD5-RLC requires ATP binding but no ATP hydrolysis [17]. Therefore, ATP binding by ATAD5-RLC allows conformational changes necessary for PCNA unloading; however, ATP hydrolysis might be needed for recycling of the Elg1-RLC [19]. 

A screen for yeast proteins that interact with the N-terminus of Elg1 resulted in a long list of proteins that either participate in the addition of SUMO (Small Ubiquitin-like Molecule), interact with SUMO, or are SUMOylated [44]. Elg1 has a SIM (SUMO interacting motif) at its N-terminus and shows affinity for SUMOylated PCNA [44]. Moreover, in mutants lacking Elg1, SUMOylated PCNA considerably accumulates on the chromatin [44,45]. Surprisingly, however, mutations in the SIM of Elg1 do not confer any obvious phenotype. Instead, mutating two threonines computationally predicted to be at the interface between PCNA and Elg1 (TT386/7) results in an intermediate phenotype between that of the WT and Elg1 deletion. A strain carrying both mutations at the SIM motif and in threonines 386-387 completely resembles an *ELG1* deletion regarding high recombination rate, accumulation of SUMOylated PCNA, and DNA damage sensitivity [45]. These results suggest that PCNA and Elg1 have two interaction points, one at or near TT386/7 and one facilitated by interaction between SUMOylated PCNA and the SIM of Elg1 [45]. Another protein that interacts with SUMOylated PCNA is Srs2 [46]. It is still unclear whether these two proteins can simultaneously bind to PCNA or compete for the interaction [35]. Interestingly, the Elg1 and Srs2 proteins have seemingly contradictory functions: while Srs2 prevents unwanted recombination by displacing Rad51 filaments [47,48], Elg1 is essential for the salvage pathway, an HR pathway that is negatively regulated by Srs2 [39]. However, they must also have overlapping roles, as *elg1Δ srs2Δ* haploids are sick, and *elg1Δ/elg1Δ srs2Δ/srs2Δ* double mutant diploids are inviable [35].

Data from eSPAN experiments suggest a bias in the amount of PCNA at the replication bubble, where more PCNA is present at the lagging strand [23]. Interestingly, DNA damage induces a remarkable switch in this bias, which leads to more PCNA accumulation on the leading strand than on the lagging strand. Two mechanisms could explain these results: the first is preferential unloading of PCNA from the lagging strand by Elg1-RLC, and the second is preferential loading of PCNA onto the leading strand. It seems that both putative mechanisms are partially responsible, as the bias shift is not observed in *elg1Δ* or *ctf18Δ* mutants [23,24]. Interestingly, these mechanisms are seemingly controlled by the DNA Damage Response (DDR, also known as DNA damage checkpoint, see below), as evidenced by the fact that deletion of either Mec1 or Rad53 (both major DNA damage kinases) also suppresses the PCNA bias shift under DNA damage conditions [23]. 

Elg1 also has a yet poorly understood role in DNA damage checkpoint activation. Elg1 becomes phosphorylated by Mec1 (ATR) in response to DNA damage at serine 112, an SQ site recognized by this protein kinase [49,50]. Mutations in this residue do not exhibit sensitivity to DNA damaging agents but show elongated telomeres (albeit not as elongated as those of *elg1Δ* strains) [50]. The DRR can be roughly separated into two pathways, the DNA damage checkpoint (DC) and the replication stress checkpoint (RC) [51]. The DC is activated by the presence of ssDNA exposed after the DNA is damaged, and by 9-1-1, a PCNA like complex, Mec1 (ATR in human) and Rad9 [52]. The RC is activated in response to fork stalling and is mediated by Mrc1 (Claspin in humans) and Mec1 as the primary mediators [53]. A role for Elg1 in the activation of DNA damage was found using yeast strains in which the DC branch or the RC branch of the DDR can be activated without actual DNA damage. By recruiting either Ddc1 and Ddc2 (DC) or Mrc1 and Ddc2 (RC) to a particular site in the genome, it is possible to elicit the appropriate checkpoint response in the absence of actual DNA damage [54,55]. Deleting Elg1, or mutating its phosphorylation site, prevents proper DC activation without affecting RC induction [56]. Similar results were exhibited by mutants carrying the *elg1-SIM+TT386/7DD* allele, which expresses a defective Elg1 protein at normal levels and produces an inactive RLC unable to unload PCNA. Thus, the effect seen in *elg1Δ* strains is not due to the lack of Elg1 protein but the lack of Elg1 ***activity***. This arguably means that the unloading of PCNA from the lagging strand by the Elg1-RLC is required to elicit the DDR, at least in this simplified system, and this may be controlled by phosphorylation of the RLC’s large subunit. However, Elg1 activity or phosphorylation is not necessary for DSB repair, suggesting that the regulation of Elg1 by phosphorylation may be restricted to particular circumstances.

### 2.3. Ctf18

Of the three RLCs, the role of Ctf18-RLC might be the most mysterious. In vitro experiments showed that this RLC possesses both unloading and loading abilities [19,57]. The Ctf18-RLC contains, in addition to the large subunit with similarity to Rfc1 (Ctf18) and the four small canonical RFC proteins, two additional subunits, Ctf8 and Dcc1 [58,59]. Mutations in *CTF18* and *CTF8* were found in genetic screens aimed at finding genes necessary for the prevention of chromosome loss or precocious sister chromatid separation (CTF stands for chromosome transition fidelity mutant) [60,61]. Mutants defective for the function of these genes exhibit sensitivity to drugs that depolarize tubulin (e.g., nocodazole and benomyl), exhibit high levels of HR [62], and share genetic interactions with genes involved in DNA replication [63]. In addition, the mutants show a precocious separation of sister chromatids, implying that Ctf18-RLC plays a role in sister chromatid cohesion (SCC) (see below). 

While there is still no clear function for Ctf8, Dcc1 has a conserved interaction with DNA polymerase Epsilon, which is in charge of DNA synthesis at the leading replication strand [64]. This interaction determines Ctf18-RLC localization [24,65,66]. Normally, Ctf18-RLC can be located at the moving replication fork, playing a role in eliciting the checkpoint response in the presence of DNA replication problems [67,68]. This localization is dependent, at least partly, on the interaction of Dcc1 with Pol Epsilon [24,65]. Mutating specific amino acids in both Pol2 (the catalytic subunit of Pol Epsilon) or residues in Dcc1 essential for the interaction with Pol Epsilon significantly reduce the localization of Ctf18-RLC to the fork [24,65]. Surprisingly, however, disrupting this interaction does not cause a precocious sister chromatid separation phenotype [24,65]. This result can mean one of two things: either the decreased localization of Ctf18 at the fork is not severe enough to cause a sister chromatid separation phenotype, or the role of Ctf18 in sister chromatid cohesion is not at the fork. This contradicts the current working model we have for the role of Ctf18 in sister chromatid establishment in which Ctf18 loads PCNA on the leading strand during replication (see the section on sister chromatid cohesion below). Ctf18 also slows down replication speed [69] and, like Elg1, affects telomere biology [34,70]. The telomeric phenotypes of mutants defective for the two RLCs are different: whereas *elg1Δ* cells show elongated telomeres, *ctf18Δ* mutants have short telomeres. The biology behind these phenotypes is still not clear and is currently being actively investigated.

Ctf18 also has a yet poorly understood role in the DNA Damage Response (DRR). Like Mrc1, Ctf18 has a role in the RC but not in the DC [71,72,73]. Thus, again the Elg1-RLC and the Ctf18-RLC seem to part ways, with the first acting in the DC and the second in the RC branch of the DDR. When cells are treated with hydroxyurea (HU), a drug that limits the production of dNTPs, thus causing fork stalling, late-firing origins of replication are prevented from initiating replication by the activity of the Rad53 kinase, part of the DRR [74]. However, the yeast strains carrying a mutation in Mrc1 or Ctf18 are defective in the activation of Rad53 and exhibit late-origin firing after exposure to HU [75]. Further, merely disrupting the interaction between Ctf18 and Pol Epsilon is sufficient to mimic the phenotype of *ctf18Δ* cells in HU, where defects in Rad53 phosphorylation result in late origin firing [71]. Therefore, apparently Ctf18-RLC carries a role in the RC that is similar to that of Mrc1 and requires localization at the fork.

Interestingly, *mrc1Δ* and *ctf18Δ* mutants show normal Rad53 phosphorylation when cells are exposed to methyl methane sulfonate (MMS), an alkylating agent [73,76]. The different phenotypes of Mrc1 have been explained [76] by arguing that HU stalls the replication fork without DNA damage, and thus short-range activation of Rad53 by Mrc1 is needed. On the other hand, instead of the one depending on Mrc1, the Rad9-mediated branch is activated in MMS-induced damage. However, the exact role of the Ctf18-RLC in the short-term activation of the DDR is still unknown. Further work is needed to understand the role of Ctf18-RLC in the DDR. 

Another striking role of Ctf18 is its phenotype in response to hyper-acetylation of histones [77]. Histone deposition on newly synthesized DNA is a complicated mechanism controlled and regulated by histone modification, mainly H3K56Ac in yeast cells [78,79]. This modification is cell-cycle-regulated, peaking at the S-phase and then removed in G2/M by the histone deacetylases Hst3 and Hst4 [80]. The double deletion of *HST3* and *HST4* leads to hyper-acetylation of all the histones, resulting in genome instability. Furthermore, the yeast cells become temperature-sensitive and dependent on the Dun1 checkpoint kinase for life [81].

Interestingly, deleting any RLCs major subunits on the background of *hst3Δ* *hst4Δ* suppresses the TS phenotype of this strain [82]. However, only deletion of *CTF18* suppresses the lethality of *dun1Δ* in the *hst3Δ hst4Δ* background [77]. Dun1 is a protein kinase that acts in the DDR after being activated by phosphorylation by the checkpoint kinase Rad53. Although Dun1 activity is usually related to the regulation of dNTP levels in the cell [83], the role of Dun1 in cells with hyper-acetylated histones is to counteract Rad53, which represses the activity of late firing origins, preventing full replication. The toxic activity of Ctf18 in cells with no Dun1 is not related to PCNA loading or unloading, but rather to its slowing-down of the replication fork: when all histones are hyper-acetylated, cells cannot distinguish between unreplicated DNA (before the moving fork, and thus usually wrapped around not acetylated histones) and already replicated DNA (behind the fork, wrapped around acetylated H3K56). Under these circumstances, Rad53 is induced, preventing firing from late origins. Dun1 prevents this activity, and in its absence, yeast cells die. Deleting *CTF18*, or preventing its attachment to Pol Epsilon, accelerates fork movement, allowing replication of regions usually covered by late-firing origins [77].

### 2.4. 9-1-1 and Rad24 (Rad17 in Humans)

Problems in the replication program can cause nicks, single-stranded gaps, or even double-stranded breaks. The cells respond to these problems by activating the DDR (DNA Damage Response) pathway. Two major, universally conserved regulatory protein kinases ATM and ATR (Tel1 and Mec1 in yeast), are activated during the DDR and they, in turn, facilitate, by phosphorylation, the activation of a cascade of additional protein kinases, such as CHK1 and CHK2 (Chk1 and Rad53 in yeast [84]), which modulate many downstream cellular pathways to cope with the DNA damage. One of the many important intermediates in the global DNA Damage Response pathway is the 9-1-1 complex (Rad9-Hus1-Rad1 in *S. pombe* and humans or Mec3-Rad17-Ddc1 in *S. cerevisiae*). The 9-1-1 is a PCNA-like complex, and, like PCNA, this ring-shaped complex encloses DNA. While PCNA is loaded by the RFC complex at 5′-3′ dsDNA-ssDNA junctions, the 9-1-1 complex is loaded by the Rad24-RLC (Rad17 in mammals) RLC at 3′-5′ dsDNA–ssDNA junctions [85,86]. Evidence shows that 9-1-1 loading is one of the first steps in DRR activation, together with Mec1 and its regulatory subunit Ddc2 [40,85]. Just artificially recruiting Ddc1 (one of the sunburnt in the 9-1-1) together with Ddc2 (the Mec1 partner) is enough to elicit the DRR [54] in the absence of DNA damage. 

The Rad24-RLC is in charge of loading the 9-1-1 ring at sites of DNA damage [86]. The *RAD24* gene is non-essential, but its inactivation causes sensitivity to DNA damage [87]. However, *rad24Δ* cells are not defective in DNA damage repair itself, but in the activation of the DDR [88,89]. 

## 3. DNA Replication

In every cell cycle, the entire DNA of a cell is unraveled and replicated. In most eukaryotic organisms, replication starts at many independent sites (origins of replication) and travels bi-directionally, forming “replicative bubbles” that eventually merge with each other. During DNA replication, DNA polymerases must copy vast quantities of DNA in a very short period. This requires high processivity; the homotrimeric ring PCNA encircles DNA and acts as a processivity factor during DNA replication. DNA is replicated asymmetrically: the ‘leading strand’ is copied somewhat continuously by DNA polymerase Epsilon (with a small role by polymerase Delta in initiation and termination [90,91]). Polymerase Delta is the main polymerase for ‘lagging strand’ synthesis [92] in a process involving the creation of many short DNA pieces (Okazaki fragments), which later become ligated. Thus, theoretically speaking, a single molecule of the PCNA ring is present on the leading strand of each replication ‘bubble’ but in many copies on the lagging strand. With that being said, empirical evidence shows that the bias in the amount of PCNA between strands is much weaker than expected [23], and recent studies suggest that this bias may not exist at all [24]. In addition, its role in holding polymerases in place, PCNA also serves as a moving platform during DNA replication, allowing DNA and chromatin-interacting proteins to operate at the fork in a DNA sequence-independent fashion [24,93]. Thus, many proteins that are important for cell viability, cell division, and genomic stability interact with PCNA, including factors involved in DNA repair, DNA replication, and chromatin remodeling activities. Many of these proteins contain PCNA-interacting peptides (PIPs) or motifs through which they interact with PCNA [44,94,95].

For the replication fork to move, it is necessary to unwind the DNA double helix. The task of unwinding DNA is accomplished by the CMG helicase, which is composed of two complexes, the MCM (composed of Mcm2-7 proteins) and the GINS (Sld5, Psf1, Psf2, and Psf3) [96], together with the Cdc45 protein. All of the proteins in the CMG are essential, while their particular role in the replication is still relatively unknown. A possible role for the GINS complex is connecting Pol Epsilon to the MCM helicase, as Psf1 physically interacts with the Dpb2 and Pol2 subunits of the polymerase [97,98]. Notably, the Pol Epsilon subunits seem to play a structural role in origin “firing” (initiating bidirectional replication). This is backed by the fact that while deletion of Pol Epsilon is lethal, a mutation in its catalytic region is not; moreover, cells carrying a truncated version of Pol Epsilon with only the region attaching it to the CMG are viable [99]. Therefore, while Pol Delta can presumably replace Pol Epsilon during the replication, the role of Pol Epsilon in origin firing is non-replaceable. 

While there is plenty of evidence for the interaction of Pol Delta and PCNA [100,101], There is hardly any evidence for the requirement of PCNA for Pol Epsilon activity in vivo. For example, there is no evidence for physical interaction between PCNA and any of Pol Epsilon subunits (Pol2, Dpb2-4) compared to numerous papers describing interactions between Pol Delta subunits and PCNA [100,101]. In fact, Pol Epsilon can replicate DNA (albeit with lower efficiency) without PCNA in vitro, contrary to Pol Delta, which shows much-reduced activity and a complete lack of processivity [102,103]. Therefore, if Pol Epsilon is tethered to the moving fork by a physical interaction with the CMG, what role exactly does PCNA serve in the leading strand? New evidence suggests its roles are related to other processes, such as chromatin formation and sister chromatid establishment (see below), which are carried out concomitantly with DNA replication.

DNA can only be synthesized in the 5′ to 3′ direction, which means that each fork is divided into a leading strand that is extended continuously in the same direction as that of the CMG helicase progress and a lagging strand that is synthesized discontinuously in a series of Okazaki fragments [104]. The replicative DNA polymerases can only extend a pre-existing strand, and therefore there is a need for a short RNA template. The Primase-Pol Alpha complex initiates replication, once on the leading strand, and for each Okazaki fragment in the lagging strand [105]. Primase begins by synthesizing an 8–10 nt RNA template which is then extended with around 10–15 nt of DNA by Pol Alpha [106]. Although Pol Alpha can create these RNA-DNA templates that initiate the replication, it is not suited for replicating the rest of the DNA, as it lacks proofreading ability and has very poor processivity [107]. Therefore, Pol Alpha is replaced by Pol Delta and Pol Epsilon to accurately and processively copy the rest of the genome [107]. RFC is in charge of stopping Pol Alpha synthesis activity, thus allowing the switch to the other polymerases. The ability of RFC to stop Pol Alpha was shown in vitro and is independent of PCNA or the replicative polymerases [108]. How does RFC know when to stop Pol Alpha in time? Switching Pol Alpha too early would leave an RNA template that Pol Delta/Epsilon could not elongate. Similarly, switching too late would lead to high mutagenesis due to the inaccurate activity of Pol Alpha. At this stage, there is no information regarding the regulation of this switch. 

While the role of PCNA in the replication of the leading strand is still not entirely clear, in the lagging strand, PCNA serves as the processivity factor for Pol Delta and coordinates the maturation of the Okazaki fragment into a continuous strand [109,110]. Okazaki fragments need to be processed soon after their synthesis, which involves removing the RNA primer and part of the DNA synthesized by Pol Alpha at the downstream Okazaki fragment [111]. This occurs by Pol Delta displacing the newly synthesized RNA-DNA strand, followed by the cutting by Rad27 (FEN1 in humans) of the flap created in the process [112]. Then, the Okazaki fragments are ligated by the replicative ligase (Cdc9 in yeast, LIG1 in humans), which also interact with PCNA [113]. After Okazaki fragment ligation, PCNA is unloaded from the chromatin by the Elg1-RLC [114].

## 4. PCNA and the DNA Damage Tolerance Pathways

**Figure 2:**
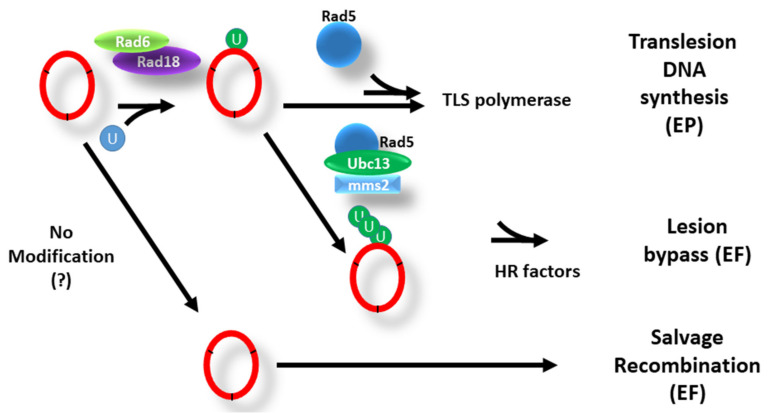
**The DDT pathways:** Representation of the four DDT (DNA Damage Tolerance) pathways. Lesions in DNA may lead to fork stalling. The damaged site can be bypassed in a mutagenic manner by recruiting an error-prone (EP) trans-lesion synthesis (TLS) polymerase. This usually requires monoubiquitination of PCNA (by Rad6/Rad18, with a still ill-understood role for Rad5), although the recruitment can be independent of PCNA modification under some circumstances. Alternatively, an error-free (EF) repair pathway is controlled by PCNA polyubiquitination, allowing a homologous recombination event using the sister chromatid that may include fork reversal. In the absence of PCNA SUMOylation, the Srs2 helicase is absent, and an error-free, homologous recombination “salvage pathway” is activated.

When DNA is damaged, PCNA turns into a signaling hub, undergoing several modifications. There are at least four different DNA damage tolerance (DDT) pathways (also known as post-replication repair or PRR) that allow bypass of lesions without their repair and are controlled directly or indirectly by PCNA modification [1] (Figure 2). Two of these lead to error-prone repair pathways, whereas two are error-free. 

The two **error-prone** repair pathways work as follows:

Regular DNA polymerases may stall in the presence of a lesion in the genome, bringing DNA replication to a halt; reinitiation of DNA replication downstream may leave a dangerous unreplicated gap behind. When these events happen, PCNA undergoes monoubiquitination at lysine 164 and recruits special DNA polymerases to replicate damaged DNA at the expense of accuracy [115]. This modification is carried out by the ubiquitin-conjugating enzyme (E2) Rad6 and the ubiquitin ligase (E3) Rad18 [116,117]. The translesion synthesis (TLS) DNA polymerases bypass the lesion to complete DNA replication at the cost of unwanted mutagenesis. PCNA monoubiquitination may serve to increase the affinity of the TLS polymerases to PCNA [118]. However, there are situations in which the error-prone polymerases are recruited without PCNA monoubiquitination [119]. It is still unclear whether, in these cases, the TLS polymerase works with PCNA and bypasses the need for its modification [120] or whether it works in a fashion that is entirely independent of PCNA. The possible explanation for the existence of two separate mechanisms of TLS polymerase recruitment can be spatial or temporal. For example, it could be that one is dominant in a specific cell cycle stage while the other works at a different cell cycle position (S-phase vs. G2, for example) or that during replication one of them acts when the fork encounters a site of DNA damage and a second mechanism is only activated at gaps left behind the fork. 

In addition, PCNA also controls two separate **error-free** repair pathways: 

DNA lesions can be bypassed by copying information from the newly replicated sister chromatid in a mechanism called template switch (TS). This requires the additional modification of the K164 monoubiquitinated PCNA with a K63-linked, non-degradative polyubiquitin chain, synthesized by the heterodimeric E2, Ubc13-Mms2, and the E3 Rad5 [121] (Figure 2). The role played by the polyubiquitin chain and the details of this mechanism are still unclear [122,123]. Currently, there are two models proposed for the mechanism of the error-free DDT: One is a template switch, in which the synthesized strand invades the recently created sister chromatid and copies the missing data from there. This mechanism may be either facilitated or regulated by polyubiquitinated PCNA. An alternative model is that PCNA polyubiquitination facilitates a fork reversal, in which the fork gets ‘pushed back’ by translocases until the newly synthesized lagging and leading strand are aligned, allowing the continuous synthesis of the arrested strand using the other newly synthesized strand as a template. Evidence for the fork reversal phenomenon comes mainly from electron microscopy studies in humans and yeast cells [124,125]. Fork reversal has also been shown in vitro, using relatively short DNA sequences [126,127,128]. Two members of the Snf2 family recognize the polyubiquitination in mammalian cells: ZRANB3 and SMARCAL1, which together with FANCM (Mph1 in yeast), and HLTF )Rad5 in yeast) reverse the fork, allowing the bypass of the damaged site in an error-free manner )reviewed in [129]). In yeast, on the other hand, there is very little evidence for such machinery. Whereas many yeast proteins belong to the SNF2 family [130], no known protein in yeast recognizes polyubiquitinated PCNA. Therefore, the precise molecular mechanism of the error-free branch of the DDT is unknown. 

Thus far, the DDT pathways appear to be positively regulated by PCNA modification. A fourth pathway is negatively regulated by SUMOylation of lysines 164 and 127 of PCNA [46]. SUMOylation of PCNA occurs typically during S-phase and after high doses of DNA damage; it requires the SUMO-specific E2 Ubc9 and the SUMO ligase Siz1 [117]. PCNA SUMOylation helps recruit Srs2, an ATP-dependent, 3′ to 5′ DNA helicase that can prevent recombination by disrupting Rad51 filaments [48,131]. Rad51, the eukaryotic ortholog of bacterial RecA [132], promotes the strand-exchange reaction during homologous recombination, and allows the single strand end of one DNA molecule to invade and pair with a homologous sequence [133]. In the absence of Srs2, whenever the fork stalls due to damage to the DNA, the ssDNA is covered by Rad51 filament, and the damaged DNA is repaired by HR in an error-free repair pathway termed the “salvage recombination pathway”. The name of this repair pathway stems from the fact that Srs2 deletion can suppress the DNA damage sensitivity of strains such as *rad6* and *rad18* to a great extent [134,135,136]. As we have seen, Elg1 is essential for the salvage pathway [39]. Despite the seemingly opposite roles for Elg1 and Srs2, they must carry at least one common task, as in the absence of both proteins, cells are sick and *elg1Δ/elg1Δ srs2Δ/srs2Δ* double mutant diploids are inviable [35].

## 5. Heterochromatin and Silencing

The DNA in all eukaryotic cells is tightly packed and has distinct three-dimensional properties. Due to the large amount of DNA, which has to fit into a tiny nucleus, the DNA has to undergo dramatic wrapping and condensation. Notably, the way a particular region in the genome is packaged regulates gene expression and affects the organism’s phenotype [137,138]. At the first level of compaction, DNA is wrapped around a nucleosome, an octamer complex consisting of a pair of each of the core histones H2A, H2B, H3, and H4 [139]. Around 150 bp of DNA is twisted around the histone octamer, resulting in ~two turns of the DNA double helix [140]. The histone linker H1 binds DNA at the entry and exit site around the histone octamer [141]. The distribution of histones along the DNA is not regular. In the heterochromatic region, the nucleosomes are tightly packed and hardly allow the binding of proteins essential for transcription. Therefore, the expression of genes in these areas is significantly reduced or silenced altogether. On the contrary, the euchromatic regions are lightly packed with nucleosomes and allow regular gene expression. Gene expression is also affected by histone modifications. The nucleosome components regulated by the plethora of post-translational modifications such as acetylation, methylation, ubiquitination, and phosphorylation has been reported. The histone modifications affect the timing and level of gene expression, and this state can be transmitted through generations by a phenomenon known as an epigenetic inheritance (reviewed in [142]).

In *S. cerevisiae*, the two primary histone post-translational modifications that control the epigenetic state are the acetylation and methylation of histone H4 or H3 [143,144]. Histone acetyltransferases (HATs) acetylate histones and make the chromatin more accessible, and histone deacetylases (HDACs) promote heterochromatin formation [145,146]. Acetylation of a histone protein removes its positive charge, thereby decreasing its interaction with other histones and the DNA itself, rending the chromatin in an ‘open’ state [147]. The Sir (silent information regulator) complex consists of Sir2, 3 and 4. Sir2, the catalytic subunit of the complex, is an NAD+-dependent deacetylase that removes acetyl modifications from histones H3 and H4 [148]. It forms a complex with Sir3 and Sir4, which preferentially bind hypo-acetylated histones [149,150,151] and thus allow their spreading along the chromatin [152]. While none of the proteins is essential for life, deleting any of them significantly reduces silencing at heterochromatic sites, and deletion of Sir2 abolishes silencing altogether [152]. While the Sir complex is the primary mechanism for heterochromatin formation, it needs to be recruited to specific sites. Various DNA silencer proteins, such as Rap1, Abf1, and some origin-recognition complex (ORC) proteins, assist in the recruitment of Sir complex to the designated sites [153]. Sir1 is a unique member of the Sir family because it is only needed for the silencing at the mating-type locus and not at the telomeres [154]. The Sir1 protein is reported to facilitate the binding of the Sir complex to the ORC proteins [155]. However, Sir1 mutants show a very mild defect in the silencing of heterochromatic regions [156]. 

DNA replication poses a severe threat to the transmission of epigenetic memory. All the nucleosomes are dislocated from the chromatin during DNA replication, and histones are randomly redistributed to the daughter strands behind the moving fork [157]. The conservation of the epigenetic memory requires that modifications present at particular histones before DNA replication should return to the same place in the two freshly replicated sister chromatids. Significant progress has been made in recent years in understanding the precise mechanism for the transmission of epigenetic memory. The coupling between replication components and several histone chaperones such as Rtt106, CAF1, Asf1, and FACT complex ensures faithful epigenetic memory transmission (reviewed in [158]).

Asf1 is a histone chaperone, which can bind to the newly synthesized H3-H4 dimers [159,160] and also has a role in histone disassembly during transcription [161]. When Asf1 is attached to a newly synthesized H3-H4, it interacts with Rtt109 (CPB in mammalian cells), a histone acetyltransferase, acetylating the newly synthesized H3 at lysine 56 [162]. Thus, H3K56 acetylation allows the distinction between old histones, transferred from the front to the back of the replication fork, and the newly deposited ones. Asf1 has a physical interaction with Rfc1, which is important for the localization of Asf1 to the fork [163]. It is still unclear if the RFC complex can bind PCNA and Asf1 simultaneously and whether there is a separation of function between these two roles of RFC. Asf1 then passes down the H3-H4 dimer to the chromatin remodelers CAF1, Rtt106, and FACT, which are in charge of inserting them in the chromatin. CAF1 is a protein complex (comprised of Cac1, Cac2, and Msi1) that acts as a histone chaperone and helps deposit H3-H4 to form a nucleosome [164]. CAF1 localizes to the fork via interactions between Cac1 and PCNA [165,166], thus coupling chromatin repacking with fork progression. CAF1 co-coordinates the nucleosome assembly with Rtt106 and the FACT complex [167,168]. Rtt106 also has a physical interaction with Elg1-RLC [169]. As with the RFC-CAF1 interaction, it is still unclear if the Elg1-RLC can bind both PCNA and Rtt106 or whether the role of Elg1 in unloading PCNA is essential for this interaction. 

After nucleosomes are re-assembled in the wake of the moving fork, Sir2-4 re-establish silencing at heterochromatic regions. Interestingly, PCNA, RFC, and Pol Epsilon play essential roles in heterochromatin establishment [170], although entry into S-phase, but not actual DNA replication, is required for the process [171,172].

Using the CRASH assay, it is possible to detect even transient loss events of epigenetic silencing at the silent mating cassettes of yeast [173]. This assay revealed that deletion of Elg1 leads to a high rate of transient loss of heterochromatin silencing [174]. This phenotype could be attributed to the role of Elg1-RLC in PCNA cycling, which is essential, in turn, for silencing [174]. Even though Elg1-RLC interacts with Rtt106 [169], the silencing defect of *elg1Δ* mutants was independent of Rtt106 or ASF1 but could be suppressed by overexpression of the CAF1 components [174]. The unloading of PCNA by the Elg1-RLC ensures that PCNA is present only at the fork, where it coordinates the activity of the histone chaperones. The increased levels of PCNA on the chromatin in *elg1Δ* mutants may titrate CAF1 components away from the actual replication sites, resulting in silencing loss.

## 6. Sister Chromatid Cohesion

The eukaryotic cells have evolved intricate mechanisms to organize the additional copy of the genome synthesized during the S-phase. Sister chromatid cohesion (SCC) is one such phenomenon where the newly replicated sister chromatids are held together by the cohesin complex until anaphase, when they eventually segregate to the opposite poles [175,176]. The physical connection between sister chromatids antagonizes the force exerted on them by the spindle-microtubule apparatus [177,178,179]. SCC is central to both the mitotic and meiotic cell divisions. Cohesin consists of four conserved proteins named Smc1, Smc3, Scc1/Mcd1 (hereafter Mcd1), and Scc3/Irr1 (hereafter Scc3) in yeast [175,180]. Smc1 and Smc3 are long (~50 nm) rod-shaped proteins, carrying two distinct terminal domains known as ATPase Head and Hinge domains, which are connected to each other through an intra-molecular coiled-coil structure [181,182]. The Kleisin subunit Mcd1 attaches to the Smc3 head domain via its N-terminus, whereas it interacts with Smc1 through its C-terminus [182,183,184]. ATP binding/hydrolysis by Smc3 and Smc1 is essential for the chromosome association of the Smc1/3 heterodimer and binding of Mcd1 to cohesin [185,186]. Scc3 binds to the Mcd1 C-terminus and is essential for cohesin integrity by a yet unclear mechanism [182]. Cohesin can adopt many different configurations, including rings, rods and butterflies [180,182]. In addition to the core subunits, several other accessory subunits such as Pds5, Scc2, Scc4, Eco1, and Wpl1 are essential for proper regulation and maintenance of cohesion [175]. Pds5 has positive and negative roles: on one hand, it is essential for cohesion maintenance and for promoting Eco1-dependent Smc3 acetylation [170,187,188]. However, it also forms a complex with cohesin release factor Wpl1 to destabilize cohesin and exclude it from chromatin [189]. 

Various models have been suggested for the mechanism by which the two sister chromatids are held together (reviewed by [190]). In principle, a cohesin complex could form a large ring, which could embrace the two sister chromatids within the central cavity. The diameter of a cohesin ring would be around ~40 nm, which is sufficient to encircle two chromatinized DNA strands and possibly large enough to allow replication machinery to pass through the ring [182,185]. In addition to the ring model, many alternative models for SCC (such as the hand-cuff model, bracelet, or butterfly models) have been proposed [191,192]. Recently, the inter-allelic complementation between defective Scc1 and Smc3 alleles has provided evidence that multiple cohesin subunits can interact with each other on the chromatin [193,194]. Indeed, many biochemical studies have confirmed that cohesin can form clusters or dimers in vivo [195,196]. The exact mechanism of action is still debated (reviewed by [190]). Cohesin holds the sister chromatids together until anaphase, when the Mcd1 subunit is cleaved by the Esp1 separase [197,198].

### Coupling of SCC Establishment with DNA Replication and the Role of RLCs

The cohesin loader complex Scc2/Scc4 loads the cohesin ring on chromatin in the G1 phase and during DNA replication [184,199]. Although cohesin can be independently loaded onto chromatin, its ability to become cohesive largely depends on DNA replication [200]. For instance, *GAL1*- induced expression of Mcd1 in the G2/M phase results in the loading of cohesin complex to chromatin; however, such cohesin cannot perform sister chromatid cohesion [201].

Eco1 is an essential protein that functions during S-phase and is vital for establishing sister chromatid cohesion. Eco1 acts as an acetyl-transferase that can acetylate itself and other cohesin components [202,203]. It acetylates conserved lysine residues (K112 and K113 in yeast, K105 and K106 in humans) on Smc3, a modification that allows stable cohesion by counteracting the Wpl1-dependent cohesin destabilization [204,205]. The *smc3-KK112,113RR* yeast strain is inviable and shows cohesion defects similar to *eco1-ts* alleles, where the cohesin is adequately loaded but fails to establish cohesion. Mutation to acetyl mimic residues (*smc3-K112Q*, *K113Q*) rescues the cohesion defects and inviability of the *eco1-ts* strain, suggesting that Smc3 is the key acetylation target of Eco1 [206,207]. Interestingly, the deletion of Wpl1 or mutations in Pds5 or Scc3 can suppress the cohesion defects associated with the temperature-sensitive (ts) allele of *eco1* [208] or the *smc3-KK112,113RR* allele [209]. Eco1 directly binds PCNA through its conserved PIP motif and relies on PCNA for its chromatin localization to allow replication-coupled cohesion establishment [210]. As expected, the acetylation of Smc3 is cell-cycle-regulated and peaks during S-phase [206]. The mammalian homologs of Eco1, Esco1, and Esco2, are also essential for SCC and rely on PCNA and the replication machinery for their recruitment to chromatin [203,211]. However, several lines of evidence suggest that Eco1 is not the sole requirement for generating cohesion, and Eco1-independent pathways for cohesion establishment likely exist [208,212,213].

**Figure 3:**
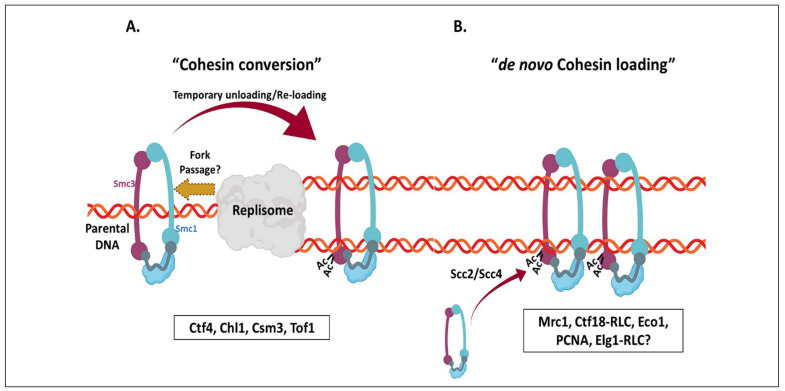
Two replication-coupled cohesion establishment pathways. (**A**) Cohesin conversion pathway: The cohesin rings already loaded onto the chromatin in late G1 phase are converted into a cohesive form in two possible ways. In the first scenario, the cohesin rings are temporarily detached from the chromatin and shifted behind the replication fork, resulting in SCC. The second scenario involves the replication fork passage through the cohesin ring. The factors essential for the cohesin conversion pathway are mentioned below the illustration. (**B**) De novo cohesin loading: The second pathway involves the loading of new cohesin rings onto the replicated sister chromatids in an Scc2/Scc4-dependent pathway. The Ctf18-RLC loads PCNA on the leading strand, which promotes Eco1 activity to allow cohesion establishment. The Elg1-RLC appears to be part of a cohesin loading pathway. However, further studies are required to understand its function in SCC.

The Ctf18-RLC plays an important role in the establishment of SCC, as the deletion of either Ctf18, Dcc1, or Ctf8 results in strong sister chromatid cohesion defects [58,59,61]. Since Ctf18, like Eco1, is present at the replisome and moves along with the replication fork [200], it may be involved directly in the loading or stabilization of cohesin. It has been suggested that the function of Ctf18-RLC in SCC is through its PCNA loading activity [24]. Ctf18-RLC loaded PCNA may serve as a landing platform for Eco1 to promote Smc3 acetylation and cohesion establishment. However, direct evidence showing the reduced chromatin recruitment of Eco1 in *ctf18Δ* mutants is still missing. Moreover, a recent study has reported that the interaction of Ctf18-RLC with Pol Epsilon is dispensable for its SCC function [24]. Various genetic screens in yeast focused on identifying synthetic genetic interactors of Ctf18 or Ctf8 revealed several bona fide replication factors vital for SCC. Among these factors, Ctf4, Csm3, Tof1, Mrc1, Chl1, and several additional replication components are required for efficient sister chromatid cohesion [214,215,216]. A careful genetic dissection of these factors resulted in the identification of two parallel cohesion establishment pathways. The Ctf18-RLC components and Mrc1 comprise one pathway, whereas the Csm3, Tof1, Chl1, and Ctf4 belong to the second pathway [217]. Whereas all the proteins are present at the replisome, Csm3 and Tof1 form a sub-complex (sometimes called the Replication Fork Protection complex) that stabilizes arrested replication forks [218,219]. Chl1, the yeast ortholog of the Fanconi Anemia gene FancJ, is a helicase of mysterious function [220,221], and Ctf4 is a component of the replisome that connects with Pol Alpha [222,223]. Mutations in each of these factors affect SCC. The current working hypothesis is that whereas Ctf18-RLC and Mrc1 affect the loading of new cohesin complexes rings at the replication fork in an Scc2/4- dependent manner [224,225], the second pathway may help convert the G1-loaded cohesin complexes to their cohesive configuration, either by facilitating the replication fork to pass through the cohesin ring or temporarily unloading and reloading cohesin behind the replication fork [224] (Figure 3). Interestingly, the *Xenopus* homologs of Ctf4 and Csm3-Tof1 also promote the establishment of sister chromatid cohesion, suggesting that these redundant genetic pathways are conserved [226].

The Elg1-RLC unloads PCNA from the lagging DNA strand during DNA replication. *ELG1* genetically interacts with several cohesin components, thus also playing a role in sister chromatid cohesion [3]. However, the molecular details of how Elg1 and PCNA regulate sister chromatid cohesion are not clear. The *elg1Δ ctf4Δ* double mutant is inviable, and overexpression of Mcd1 or the cohesin loader Scc2 rescues the synthetic lethality [227], suggesting that the inviability is due to lack of proper SCC. Further analysis showed that *elg1Δ* mutants exhibit mild SCC defects, by affecting the Ctf18-mediated pathway [227]. Thus, a *ctf4Δ elg1Δ* double mutant is defective for the two cohesion pathways described above. Surprisingly, however, deletion of *ELG1* can rescue the temperature sensitivity and sister chromatid cohesion defects of Pds5 and Eco1 temperature-sensitive strains. Consistently, Elg1 deletion exacerbates the phenotype of *mcd1-ts* or *smc3-ts* alleles [228,229]. Therefore, it appears that Elg1-RLC promotes sister chromatid cohesion; however, further studies are needed to understand its complex genetic interactions [230]. A recent study claims that Elg1 deletion suppresses the sister chromatid cohesion defect of *ctf18Δ*, because presumably the defect in these cells is due to lower PCNA levels (and thus presumably lower Eco1 activity), and deletion of the PCNA unloader *ELG1* can restore enough PCNA to ensure proper SCC [24]. This is in contradiction to previous results that failed to see a suppression of *ctf18* by deleting *ELG1*. Instead, the SCC defects observed in the double mutant were similar to those of the most defective mutant, *ctf18Δ*, implying that the two RLCs work in a single pathway [227]. Further studies are required to better understand the relationship between these RLCs and their roles in SCC.

## 7. Summary

By playing a central role in DNA replication and repair, the PCNA clamp and its loaders/unloaders affect almost any aspect of nuclear function. We summarized current knowledge about the role of these proteins in DNA replication, DNA repair, DNA Damage Response, chromatin configuration, and sister chromatid cohesion. Much is still left to be discovered. Given the conservation of all the systems described throughout eukaryotic evolution, the knowledge gained in yeast is likely to be valid for other organisms, including humans.

## Data Availability

No new data were created or analyzed in this study. Data sharing is not applicable to this article.

## References

[1] Arbel M., Liefshitz B., Kupiec M. (2020). DNA damage bypass pathways and their effect on mutagenesis in yeast. FEMS Microbiol. Rev..

[2] Boiteux S., Jinks-Robertson S. (2013). DNA repair mechanisms and the bypass of DNA damage in Saccharomyces cerevisiae. Genetics.

[3] Kupiec M. (2016). Alternative clamp loaders/unloaders. FEMS Yeast Res..

[4] Lee K.Y., Park S.H. (2020). Eukaryotic clamp loaders and unloaders in the maintenance of genome stability. Exp. Mol. Med..

[5] Ohashi E., Tsurimoto T. (2017). Functions of multiple clamp and clamp-loader complexes in eukaryotic DNA replication. Adv. Exp. Med. Biol..

[6] Shiomi Y., Nishitani H. (2017). Control of genome integrity by RFC complexes; conductors of PCNA loading onto and unloading from chromatin during DNA replication. Genes.

[7] Majka J., Burgers P.M.J. (2004). The PCNA-RFC Families of DNA Clamps and Clamp Loaders. Prog. Nucleic Acid Res. Mol. Biol..

[8] Sakato M., Zhou Y., Hingorani M.M. (2012). ATP binding and hydrolysis-driven rate-determining events in the RFC-catalyzed PCNA clamp loading reaction. J. Mol. Biol..

[9] Dionne I., Brown N.J., Woodgate R., Bell S.D. (2008). On the mechanism of loading the PCNA sliding clamp by RFC. Mol. Microbiol..

[10] Cullmann G., Fien K., Kobayashi R., Stillman B. (1995). Characterization of the five replication factor C genes of Saccharomyces cerevisiae. Mol. Cell. Biol..

[11] Mossi R., Hübscher U. (1998). Clamping down on clamps and clamp loaders—The eukaryotic replication factor C. Eur. J. Biochem..

[12] Bunz F., Kobayashi R., Stillman B. (1993). cDNAs encoding the large subunit of human replication factor C. Proc. Natl. Acad. Sci. USA.

[13] Uhlmann F., Cai J., Flores-Rozas H., Dean F.B., Finkelstein J., O’Donnell M., Hurwitz J. (1996). In vitro reconstitution of human replication factor C from its five subunits. Proc. Natl. Acad. Sci. USA.

[14] Bowman G.D., O’Donnell M., Kuriyan J. (2004). Structural analysis of a eukaryotic sliding DNA clamp-clamp loader complex. Nature.

[15] Gomes X.V., Gary S.L., Burgers P.M.J. (2000). Overproduction in *Escherichia coli* and characterization of yeast replication factor C lacking the ligase homology domain. J. Biol. Chem..

[16] Yao N.Y., O’Donnell M. (2012). The RFC clamp loader: Structure and Function. Subcell. Biochem..

[17] Gaubitz C., Liu X., Magrino J., Stone N.P., Landeck J., Hedglin M., Kelch B.A. (2020). Structure of the human clamp loader reveals an autoinhibited conformation of a substrate-bound AAA+ switch. Proc. Natl. Acad. Sci. USA.

[18] Kelch B.A. (2016). Review: The lord of the rings: Structure and mechanism of the sliding clamp loader. Biopolymers.

[19] Kang M.S., Ryu E., Lee S.W., Park J., Ha N.Y., Ra J.S., Kim Y.J., Kim J., Abdel-Rahman M., Park S.H. (2019). Regulation of PCNA cycling on replicating DNA by RFC and RFC-like complexes. Nat. Commun..

[20] Hedglin M., Aitha M., Benkovic S.J. (2017). Monitoring the Retention of Human Proliferating Cell Nuclear Antigen at Primer/Template Junctions by Proteins That Bind Single-Stranded DNA. Biochemistry.

[21] Hedglin M., Perumal S.K., Hu Z., Benkovic S. (2013). Stepwise assembly of the human replicative polymerase holoenzyme. Elife.

[22] Ben-Aroya S., Koren A., Liefshitz B., Steinlauf R., Kupiec M. (2003). ELG1, a yeast gene required for genome stability, forms a complex related to replication factor C. Proc. Natl. Acad. Sci. USA.

[23] Yu C., Gan H., Han J., Zhou Z.X., Jia S., Chabes A., Farrugia G., Ordog T., Zhang Z. (2014). Strand-Specific Analysis Shows Protein Binding at Replication Forks and PCNA Unloading from Lagging Strands when Forks Stall. Mol. Cell.

[24] Liu H.W., Bouchoux C., Panarotto M., Kakui Y., Patel H., Uhlmann F. (2020). Division of Labor between PCNA Loaders in DNA Replication and Sister Chromatid Cohesion Establishment. Mol. Cell.

[25] Lee B.-G., Roig M.B., Jansma M., Petela N., Metson J., Nasmyth K., Löwe J., Johnson C., Gali V.K.V.K., Takahashi T.S. (2016). ELG1, a yeast gene required for genome stability, forms a complex related to replication factor C. Mol. Cell.

[26] Johnson C., Gali V.K., Takahashi T.S., Kubota T. (2016). PCNA Retention on DNA into G2/M Phase Causes Genome Instability in Cells Lacking Elg1. Cell Rep..

[27] Kubota T., Nishimura K., Kanemaki M.T., Donaldson A.D. (2013). The Elg1 Replication Factor C-like Complex Functions in PCNA Unloading during DNA Replication. Mol. Cell.

[28] Scholes D.T., Banerjee M., Bowen B., Curcio M.J. (2001). Multiple regulators of Ty1 transposition in Saccharomyces cerevisiae have conserved roles in genome maintenance. Genetics.

[29] Bellaoui M., Chang M., Ou J., Xu H., Boone C., Brown G.W. (2003). Elg1 forms an alternative RFC complex important for DNA replication and genome integrity. EMBO J..

[30] Huang M.-E., Riot A.-G., Nicolas A., Kolodner R.D. (2003). A genomewide screen in Saccharomyces cerevisiae for genes that suppress the accumulation of mutations. Proc. Natl. Acad. Sci. USA.

[31] Smith S., Hwang J.-Y., Banerjee S., Majeed A., Gupta A., Myung K. (2004). Mutator genes for suppression of gross chromosomal rearrangements identified by a genome wide screening in Saccharomyces cerevisiae. Proc. Natl. Acad. Sci. USA.

[32] Smolikov S., Mazor Y., Krauskopf A. (2004). ELG1, a regulator of genome stability, has a role in telomere length regulation and in silencing. Proc. Natl. Acad. Sci. USA.

[33] Ogiwara H., Ui A., Enomoto T., Seki M. (2007). Role of Elg1 protein in double strand break repair. Nucleic Acids Res..

[34] Askree S.H., Yehuda T., Smolikov S., Gurevich R., Hawk J., Coker C., Krauskopf A., Kupiec M., McEachern M.J. (2004). A genome-wide screen for Saccharomyces cerevisiae deletion mutants that affect telomere length. Proc. Natl. Acad. Sci. USA.

[35] Gazy I., Liefshitz B., Bronstein A., Parnas O., Atias N., Sharan R., Kupiec M. (2013). A genetic screen for high copy number suppressors of the synthetic lethality between *elg1Δ* and *srs2Δ* in yeast. G3 Genes Genomes Genet..

[36] Gazy I., Liefshitz B., Parnas O., Kupiec M. (2015). Elg1, a central player in genome stability. Mutat. Res. Rev. Mutat. Res..

[37] Davidson M.B., Brown G.W. (2008). The N- and C-termini of Elg1 contribute to the maintenance of genome stability. DNA Repair. (Amst)..

[38] Alvaro D., Lisby M., Rothstein R. (2007). Genome-wide analysis of Rad52 foci reveals diverse mechanisms impacting recombination. PLoS Genet..

[39] Arbel M., Bronstein A., Sau S., Liefshitz B., Kupiec M. (2020). Access to pcna by srs2 and elg1 controls the choice between alternative repair pathways in saccharomyces cerevisiae. MBio.

[40] Bylund G.O., Majka J., Burgers P.M.J. (2006). Overproduction and Purification of RFC-Related Clamp Loaders and PCNA-Related Clamps from *Saccharomyces cerevisiae*. Methods in Enzymology.

[41] Ho B., Baryshnikova A., Brown G.W. (2018). Unification of Protein Abundance Datasets Yields a Quantitative Saccharomyces cerevisiae Proteome. Cell Syst..

[42] Kang S., Warner M.D., Bell S.P. (2018). Multiple Functions for Mcm2-7 ATPase Motifs during Replication Initiation. Mol. Cell.

[43] Kubota T., Myung K., Donaldson A.D. (2014). Is PCNA unloading the central function of the Elg1/ ATAD5 replication factor C-like complex?. Cell Cycle.

[44] Parnas O., Zipin-Roitman A., Pfander B., Liefshitz B., Mazor Y., Ben-Aroya S., Jentsch S., Kupiec M. (2010). Elg1, an alternative subunit of the RFC clamp loader, preferentially interacts with SUMOylated PCNA. EMBO J..

[45] Shemesh K., Sebesta M., Pacesa M., Sau S., Bronstein A., Parnas O., Liefshitz B., Venclovas Č., Krejci L., Kupiec M. (2017). A structure-function analysis of the yeast Elg1 protein reveals the importance of PCNA unloading in genome stability maintenance. Nucleic Acids Res..

[46] Pfander B., Moldovan G.-L., Sacher M., Hoege C., Jentsch S. (2005). SUMO-modified PCNA recruits Srs2 to prevent recombination during S phase. Nature.

[47] Burkovics P., Sebesta M., Sisakova A., Plault N., Szukacsov V., Robert T., Pinter L., Marini V., Kolesar P., Haracska L. (2013). Srs2 mediates PCNA-SUMO-dependent inhibition of DNA repair synthesis. EMBO J..

[48] Veaute X., Jeusset J., Soustelle C., Kowalczykowski S.C., Le Cam E., Fahre F. (2003). The Srs2 helicase prevents recombination by disrupting Rad51 nucleoprotein filaments. Nature.

[49] Lanz M.C., Yugandhar K., Gupta S., Sanford E., Faça V., Vega S., Joiner A., Fromme C., Yu H., Smolka M.B. (2019). In-depth and 3-dimensional exploration of the budding yeast phosphoproteome. bioRxiv.

[50] Shkedy D., Singh N., Shemesh K., Amir A., Geiger T., Liefshitz B., Harari Y., Kupiec M. (2015). Regulation of Elg1 activity by phosphorylation. Cell Cycle.

[51] Pardo B., Crabbé L., Pasero P. (2017). Signaling pathways of replication stress in yeast. FEMS Yeast Res..

[52] Toh G.W.L., Lowndes N.F. (2003). Role of the Saccharomyces cerevisiae Rad9 protein in sensing and responding to DNA damage. Biochem. Soc. Trans..

[53] Alcasabas A.A., Osborn A.J., Bachant J., Hu F., Werler P.J.H., Bousset K., Furuya K., Diffley J.F.X., Carr A.M., Elledge S.J. (2001). Mrc1 transduces signals of DNA replication stress to activate Rad53. Nat. Cell Biol..

[54] Bonilla C.Y., Melo J.A., Toczyski D.P. (2008). Colocalization of Sensors Is Sufficient to Activate the DNA Damage Checkpoint in the Absence of Damage. Mol. Cell.

[55] Berens T.J., Toczyski D.P. (2012). Colocalization of Mec1 and Mrc1 is sufficient for Rad53 phosphorylation in vivo. Mol. Biol. Cell.

[56] Sau S., Liefshitz B., Kupiec M. (2019). The yeast pcna unloader Elg1 RFC-like complex plays a role in eliciting the DNA damage checkpoint. MBio.

[57] Bylund G.O., Burgers P.M.J. (2005). Replication protein A-directed unloading of PCNA by the Ctf18 cohesion establishment complex. Mol. Cell. Biol..

[58] Hanna J.S., Kroll E.S., Lundblad V., Spencer F.A. (2001). *Saccharomyces cerevisiae* CTF18 and CTF4 are required for sister chromatid cohesion. Mol. Cell. Biol..

[59] Mayer M.L., Gygi S.P., Aebersold R., Hieter P. (2001). Identification of RFC(Ctf18p, Ctf8p, Dcc1p): An alternative RFC complex required for sister chromatid cohesion in *S. cerevisiae*. Mol. Cell.

[60] Spencer F., Gerring S.L., Connelly C., Hieter P. (1990). Mitotic chromosome transmission fidelity mutants in *Saccharomyces cerevisiae*. Genetics.

[61] Kouprina N., Tsouladze A., Koryabin M., Hieter P., Spencer F., Larionov V. (1993). Identification and genetic mapping of CHL genes controlling mitotic chromosome transmission in yeast. Yeast.

[62] Kouprina N., Kroll E., Kirillov A., Bannikov V., Zakharyev V., Larionov V. (1994). CHL12, a gene essential for the fidelity of chromosome transmission in the yeast *Saccharomyces cerevisiae*. Genetics.

[63] Miles J., Formosa T. (1992). Evidence that POB1, a *Saccharomyces cerevisiae* protein that binds to DNA polymerase α, acts in DNA metabolism in vivo. Mol. Cell. Biol..

[64] Pursell Z.F., Isoz I., Lundström E.-B., Johansson E., Kunkel T.A. (2007). Yeast DNA polymerase ε participates in leading-strand DNA replication. Science.

[65] Stokes K., Winczura A., Song B., De Piccoli G., Grabarczyk D.B. (2020). Ctf18-RFC and DNA Pol ϵ form a stable leading strand polymerase/clamp loader complex required for normal and perturbed DNA replication. Nucleic Acids Res..

[66] Wade B.O., Liu H.W., Samora C.P., Uhlmann F., Singleton M.R. (2017). Structural studies of RFC C tf18 reveal a novel chromatin recruitment role for Dcc1. EMBO Rep..

[67] Crabbé L., Thomas A., Pantesco V., De Vos J., Pasero P., Lengronne A. (2010). Analysis of replication profiles reveals key role of RFC-Ctf18 in yeast replication stress response. Nat. Struct. Mol. Biol..

[68] Kubota T., Hiraga S.-I., Yamada K., Lamond A.I., Donaldson A.D. (2011). Quantitative proteomic analysis of chromatin reveals that Ctf18 acts in the DNA replication checkpoint. Mol. Cell. Proteomics.

[69] Terret M.-E., Sherwood R., Rahman S., Qin J., Jallepalli P.V. (2009). Cohesin acetylation speeds the replication fork. Nature.

[70] Hiraga S.-I., Robertson E.D., Donaldson A.D. (2006). The Ctf18 RFC-like complex positions yeast telomeres but does not specify their replication time. EMBO J..

[71] García-Rodríguez L.J., De Piccoli G., Marchesi V., Jones R.C., Edmondson R.D., Labib K. (2015). A conserved Polϵ binding module in Ctf18-RFC is required for S-phase checkpoint activation downstream of Mec1. Nucleic Acids Res..

[72] Poli J., Tsaponina O., Crabbé L., Keszthelyi A., Pantesco V., Chabes A., Lengronne A., Pasero P. (2012). dNTP pools determine fork progression and origin usage under replication stress. EMBO J..

[73] Ogiwara H., Ohuchi T., Ui A., Tada S., Enomoto T., Seki M. (2007). Ctf18 is required for homologous recombination-mediated double-strand break repair. Nucleic Acids Res..

[74] Tercero J.A., Diffley J.F.X. (2001). Regulation of DNA replication fork progression through damaged DNA by the Mec1/Rad53 checkpoint. Nature.

[75] Santocanale C., Diffley J.F.X. (1998). A Mec1-and Rad53-dependent checkpoint controls late-firing origins of DNA replication. Nature.

[76] Bacal J., Moriel-Carretero M., Pardo B., Barthe A., Sharma S., Chabes A., Lengronne A., Pasero P. (2018). Mrc1 and Rad9 cooperate to regulate initiation and elongation of DNA replication in response to DNA damage. EMBO J..

[77] Gershon L., Kupiec M. (2021). A novel role for Dun1 in the regulation of origin firing upon hyper-acetylation of H3K56. PLoS Genet..

[78] Topal S., Vasseur P., Radman-Livaja M., Peterson C.L. (2019). Distinct transcriptional roles for Histone H3-K56 acetylation during the cell cycle in Yeast. Nat. Commun..

[79] Kaplan T., Liu C.L., Erkmann J.A., Holik J., Grunstein M., Kaufman P.D., Friedman N., Rando O.J. (2008). Cell cycle- and chaperone-mediated regulation of H3K56ac incorporation in yeast. PLoS Genet..

[80] Masumoto H., Hawke D., Kobayashi R., Verreault A. (2005). A role for cell-cycle-regulated histone H3 lysine 56 acetylation in the DNA damage response. Nature.

[81] Celic I., Masumoto H., Griffith W.P., Meluh P., Cotter R.J., Boeke J.D., Verreault A. (2006). The Sirtuins Hst3 and Hst4p Preserve Genome Integrity by Controlling Histone H3 Lysine 56 Deacetylation. Curr. Biol..

[82] Celic I., Verreault A., Boeke J.D. (2008). Histone H3 K56 hyperacetylation perturbs replisomes and causes DNA damage. Genetics.

[83] Zhao X., Rothstein R. (2002). The Dun1 checkpoint kinase phosphorylates and regulates the ribonucleotide reductase inhibitor Sml1. Proc. Natl. Acad. Sci. USA.

[84] Lanz M.C., Dibitetto D., Smolka M.B. (2019). DNA damage kinase signaling: Checkpoint and repair at 30 years. EMBO J..

[85] Majka J., Binz S.K., Wold M.S., Burgers P.M.J. (2006). Replication protein a directs loading of the DNA damage checkpoint clamp to 5′-DNA junctions. J. Biol. Chem..

[86] Majka J., Burgers P.M.J. (2003). Yeast Rad17/Mec3/Ddc1: A sliding clamp for the DNA damage checkpoint. Proc. Natl. Acad. Sci. USA.

[87] Eckardt-Schupp F., Siede W., Game J.C. (1987). The RAD24 (= R(S1)) gene product of *Saccharomyces cerevisiae* participates in two different pathways of DNA repair. Genetics.

[88] Aylon Y., Kupiec M. (2003). The Checkpoint Protein Rad24 of *Saccharomyces cerevisiae* Is Involved in Processing Double-Strand Break Ends and in Recombination Partner Choice. Mol. Cell. Biol..

[89] De la Torre-Ruiz M.A., Green C.M., Lowndes N.F. (1998). RAD9 and RAD24 define two additive, interacting branches of the DNA damage checkpoint pathway in budding yeast normally required for Rad53 modification and activation. EMBO J..

[90] Zhou Z.X., Lujan S.A., Burkholder A.B., Garbacz M.A., Kunkel T.A. (2019). Roles for DNA polymerase δ in initiating and terminating leading strand DNA replication. Nat. Commun..

[91] Johnson R.E., Klassen R., Prakash L., Prakash S. (2015). A Major Role of DNA Polymerase δ in Replication of Both the Leading and Lagging DNA Strands. Mol. Cell.

[92] Georgescu R.E., Schauer G.D., Yao N.Y., Langston L.D., Yurieva O., Zhang D., Finkelstein J., O’Donnell M.E. (2015). Reconstitution of a eukaryotic replisome reveals suppression mechanisms that define leading/lagging strand operation. Elife.

[93] Paunesku T., Mittal S., Protić M., Oryhon J., Korolev S.V., Joachimiak A., Woloschak G.E. (2001). Proliferating cell nuclear antigen (PCNA): Ringmaster of the genome. Int. J. Radiat. Biol..

[94] Moldovan G.-L., Pfander B., Jentsch S. (2007). PCNA, the Maestro of the Replication Fork. Cell.

[95] Maga G., Hübscher U. (2003). Proliferating cell nuclear antigen (PCNA): A dancer with many partners. J. Cell Sci..

[96] Takayama Y., Kamimura Y., Okawa M., Muramatsu S., Sugino A., Araki H. (2003). GINS, a novel multiprotein complex required for chromosomal DNA replication in budding yeast. Genes Dev..

[97] Sengupta S., Van Deursen F., De Piccoli G., Labib K. (2013). Dpb2 Integrates the Leading-Strand DNA Polymerase into the Eukaryotic Replisome. Curr. Biol..

[98] Langston L.D., Zhang D., Yurieva O., Georgescu R.E., Finkelstein J., Yao N.Y., Indiani C., O’Donnell M.E. (2014). CMG helicase and DNA polymerase ε form a functional 15-subunit holoenzyme for eukaryotic leading-strand DNA replication. Proc. Natl. Acad. Sci. USA.

[99] Stepchenkova E.I., Zhuk A.S., Cui J., Tarakhovskaya E.R., Barbari S.R., Shcherbakova P.V., Polev D.E., Fedorov R., Poliakov E., Rogozin I.B. (2021). Compensation for the absence of the catalytically active half of DNA polymerase ε in yeast by positively selected mutations in CDC28. Genetics.

[100] Johansson E., Garg P., Burgers P.M.J. (2004). The Pol32 Subunit of DNA Polymerase δ Contains Separable Domains for Processive Replication and Proliferating Cell Nuclear Antigen (PCNA) Binding. J. Biol. Chem..

[101] Krogan N.J., Cagney G., Yu H., Zhong G., Guo X., Ignatchenko A., Li J., Pu S., Datta N., Tikuisis A.P. (2006). Global landscape of protein complexes in the yeast *Saccharomyces cerevisiae*. Nature.

[102] Burgers P.M.J. (1991). *Saccharomyces cerevisiae* replication factor C. II. Formation and activity of complexes with the proliferating cell nuclear antigen and with DNA polymerases delta and epsilon. J. Biol. Chem..

[103] Yuan Z., Georgescu R., Schauer G.D., O’Donnell M.E., Li H. (2020). Structure of the polymerase ε holoenzyme and atomic model of the leading strand replisome. Nat. Commun..

[104] Okazaki R., Okazaki T., Sakabe K., Sugimoto K., Sugino A. (1968). Mechanism of DNA chain growth. I. Possible discontinuity and unusual secondary structure of newly synthesized chains. Proc. Natl. Acad. Sci. USA.

[105] Sugino A. (1995). Yeast DNA polymerases and their role at the replication fork. Trends Biochem. Sci..

[106] Perera R.L., Torella R., Klinge S., Kilkenny M.L., Maman J.D., Pellegrini L. (2013). Mechanism for priming DNA synthesis by yeast DNA Polymerase α. Elife.

[107] Bebenek K., Kunkel T.A. (2004). Functions of DNA polymerases. Adv. Protein Chem..

[108] Maga G., Stucki M., Spadari S., Hübscher U. (2000). DNA polymerase switching: I. Replication factor C displaces DNA polymerase α prior to PCNA loading. J. Mol. Biol..

[109] Bauer G.A., Burgers P.M.J. (1988). The yeast analog of mammalian cyclin/proliferating-cell nuclear antigen interacts with mammalian DNA polymerase δ. Proc. Natl. Acad. Sci. USA.

[110] Sporbert A., Domaing P., Leonhardt H., Cardoso M.C. (2005). PCNA acts as a stationary loading platform for transiently interacting Okazaki fragment maturation proteins. Nucleic Acids Res..

[111] Pavlov Y.I., Frahm C., McElhinny S.A.N., Niimi A., Suzuki M., Kunkel T.A. (2006). Evidence that errors made by DNA polymerase α are corrected by DNA polymerase δ. Curr. Biol..

[112] Stodola J.L., Burgers P.M. (2016). Resolving individual steps of Okazaki-fragment maturation at a millisecond timescale. Nat. Struct. Mol. Biol..

[113] Johnston L.H. (1983). The cdc9 ligase joins completed replicons in Baker’s yeast. MGG Mol. Gen. Genet..

[114] Kubota T., Katou Y., Nakato R., Shirahige K., Donaldson A.D. (2015). Replication-Coupled PCNA Unloading by the Elg1 Complex Occurs Genome-wide and Requires Okazaki Fragment Ligation. Cell Rep..

[115] Guilliam T.A., Yeeles J.T.P. (2020). Reconstitution of translesion synthesis reveals a mechanism of eukaryotic DNA replication restart. Nat. Struct. Mol. Biol..

[116] Bailly V., Lauder S., Prakash S., Prakash L. (1997). Yeast DNA repair proteins Rad6 and Rad18 form a heterodimer that has ubiquitin conjugating, DNA binding, and ATP hydrolytic activities. J. Biol. Chem..

[117] Hoege C., Pfander B., Moldovan G.-L., Pyrowolakis G., Jentsch S. (2002). RAD6-dependent DNA repair is linked to modification of PCNA by ubiquitin and SUMO. Nature.

[118] Bienko M., Green C.M., Crosetto N., Rudolf F., Zapart G., Coull B., Kannouche P., Wider G., Peter M., Lehmann A.R. (2005). Biochemistry: Ubiquitin-binding domains in Y-family polymerases regulate translesion synthesis. Science.

[119] Brocas C., Charbonnier J.B., Dhérin C., Gangloff S., Maloisel L. (2010). Stable interactions between DNA polymerase δ catalytic and structural subunits are essential for efficient DNA repair. DNA Repair.

[120] Tellier-Lebegue C., Dizet E., Ma E., Veaute X., Coïc E., Charbonnier J.B., Maloisel L. (2017). The translesion DNA polymerases Pol ζ and Rev1 are activated independently of PCNA ubiquitination upon UV radiation in mutants of DNA polymerase δ. PLoS Genet..

[121] Ulrich H.D., Jentsch S. (2000). Two RING finger proteins mediate cooperation between ubiquitin-conjugating enzymes in DNA repair. EMBO J..

[122] Branzei D., Vanoli F., Foiani M. (2008). SUMOylation regulates Rad18-mediated template switch. Nature.

[123] Branzei D., Psakhye I. (2016). DNA damage tolerance. Curr. Opin. Cell Biol..

[124] Giannattasio M., Zwicky K., Follonier C., Foiani M., Lopes M., Branzei D. (2014). Visualization of recombination-mediated damage bypass by template switching. Nat. Struct. Mol. Biol..

[125] Blastyák A., Pintér L., Unk I., Prakash L., Prakash S., Haracska L. (2007). Yeast Rad5 Protein Required for Postreplication Repair Has a DNA Helicase Activity Specific for Replication Fork Regression. Mol. Cell.

[126] Vujanovic M., Krietsch J., Raso M.C., Terraneo N., Zellweger R., Schmid J.A., Taglialatela A., Huang J.W., Holland C.L., Zwicky K. (2017). Replication Fork Slowing and Reversal upon DNA Damage Require PCNA Polyubiquitination and ZRANB3 DNA Translocase Activity. Mol. Cell.

[127] Kolinjivadi A.M., Sannino V., De Antoni A., Zadorozhny K., Kilkenny M., Técher H., Baldi G., Shen R., Ciccia A., Pellegrini L. (2017). Smarcal1-Mediated Fork Reversal Triggers Mre11-Dependent Degradation of Nascent DNA in the Absence of Brca2 and Stable Rad51 Nucleofilaments. Mol. Cell.

[128] Vaisman A., Tissier A., Frank E.G., Goodman M.F., Woodgate R. (2001). Human DNA Polymerase ι Promiscuous Mismatch Extension. J. Biol. Chem..

[129] Quinet A., Lemaçon D., Vindigni A. (2017). Replication Fork Reversal: Players and Guardians. Mol. Cell.

[130] Joseph S.A., Taglialatela A., Leuzzi G., Huang J.W., Cuella-Martin R., Ciccia A. (2020). Time for remodeling: SNF2-family DNA translocases in replication fork metabolism and human disease. DNA Repair.

[131] Fabre F., Chan A., Heyer W.D., Gangloff S. (2002). Alternate pathways involving Sgs1/Top3, Mus81/ Mms4, and Srs2 prevent formation of toxic recombination intermediates from single-stranded gaps created by DNA replication. Proc. Natl. Acad. Sci. USA.

[132] Shinohara A., Ogawa H., Ogawa T. (1992). Rad51 protein involved in repair and recombination in *S. cerevisiae* is a RecA-like protein. Cell.

[133] Sung P. (1994). Catalysis of ATP-dependent homologous DNA pairing and strand exchange by yeast RAD51 protein. Science.

[134] Ulrich H.D. (2001). The srs2 suppressor of UV sensitivity acts specifically on the RAD5- and MMS2-dependent branch of the RAD6 pathway. Nucleic Acids Res..

[135] Aboussekhra A., Chanet R., Zgaga Z., Cassier-Chauvat C., Heude M., Fabre F. (1989). RADH, a gene of *Saccharomyces cerevisiae* encoding a putative DNA helicase involved in DNA repair. Characteristics of radH mutants and sequence of the gene. Nucleic Acids Res..

[136] Rong L., Palladino F., Aguilera A., Klein H.L. (1991). The hyper-gene conversion hpr5-1 mutation of *Saccharomyces cerevisiae* is an allele of the SRS2/RADH gene. Genetics.

[137] Yuan G.C., Liu Y.J., Dion M.F., Slack M.D., Wu L.F., Altschuler S.J., Rando O.J. (2005). Molecular biology: Genome-scale identification of nucleosome positions in *S. cerevisiae*. Science.

[138] Anderson J.D., Widom J. (2000). Sequence and position-dependence of the equilibrium accessibility of nucleosomal DNA target sites. J. Mol. Biol..

[139] Schäfer G., Smith E.M., Patterton H.G. (2005). The Saccharomyces cerevisiae linker histone Hho1p, with two globular domains, can simultaneously bind to two four-way junction DNA molecules. Biochemistry.

[140] Richmond T.J., Davey C.A. (2003). The structure of DNA in the nucleosome core. Nature.

[141] Ushinsky S.C., Bussey H., Ahmed A.A., Wang Y., Friesen J., Williams B.A., Storms R.K. (1997). Histone H1 in *Saccharomyces cerevisiae*. Yeast.

[142] Lacal I., Ventura R. (2018). Epigenetic Inheritance: Concepts, Mechanisms and Perspectives. Front. Mol. Neurosci..

[143] Kayne P.S., Kim U.J., Han M., Mullen J.R., Yoshizaki F., Grunstein M. (1988). Extremely conserved histone H4 N terminus is dispensable for growth but essential for repressing the silent mating loci in yeast. Cell.

[144] Laurenson P., Rine J. (1992). Silencers, silencing, and heritable transcriptional states. Microbiol. Rev..

[145] Brownell J.E., Zhou J., Ranalli T., Kobayashi R., Edmondson D.G., Roth S.Y., Allis C.D. (1996). Tetrahymena histone acetyltransferase A: A homolog to yeast Gcn5p linking histone acetylation to gene activation. Cell.

[146] Nakajima H. (2007). A mammalian histone deacetylase related to the yeast transcriptional regulator Rpd3p. Tanpakushitsu Kakusan Koso.

[147] Shogren-Knaak M., Ishii H., Sun J.M., Pazin M.J., Davie J.R., Peterson C.L. (2006). Histone H4-K16 acetylation controls chromatin structure and protein interactions. Science.

[148] Imai S.-I., Armstrong C.M., Kaeberlein M., Guarente L. (2000). Transcriptional silencing and longevity protein Sir2 is an NAD-dependent histone deacetylase. Nature.

[149] Hoppe G.J., Tanny J.C., Rudner A.D., Gerber S.A., Danaie S., Gygi S.P., Moazed D. (2002). Steps in Assembly of Silent Chromatin in Yeast: Sir3-Independent Binding of a Sir2/Sir4 Complex to Silencers and Role for Sir2-Dependent Deacetylation. Mol. Cell. Biol..

[150] Luo K., Vega-Palas M.A., Grunstein M. (2002). Rap1-Sir4 binding independent of other Sir, yKu, or histone interactions initiates the assembly of telomeric heterochromatin in yeast. Genes Dev..

[151] Rusche L.N., Kirchmaier A.L., Rine J. (2002). Ordered Nucleation and Spreading of Silenced Chromatin in *Saccharomyces cerevisiae*. Mol. Biol. Cell.

[152] Pillus L., Rine J. (1989). Epigenetic inheritance of transcriptional states in *S. cerevisiae*. Cell.

[153] Morse R.H. (2000). RAP, RAP, open up! New wrinkles for RAP1 in yeast. Trends Genet..

[154] Aparicio O.M., Billington B.L., Gottschling D.E. (1991). Modifiers of position effect are shared between telomeric and silent mating-type loci in *S. cerevisiae*. Cell.

[155] Gardner K.A., Rine J., Fox C.A. (1999). A Region of the Sir1 Protein Dedicated to Recognition of a Silencer and Required for Interaction with the Orc1 Protein in *Saccharomyces cerevisiae*. Genetics.

[156] Pillus L., Rine J. (2004). SIR1 and the origin of epigenetic states in *Saccharomyces cerevisiae*. Cold Spring Harb. Symp. Quant. Biol..

[157] Jackson V. (1988). Deposition of newly synthesized histones: Hybrid nucleosomes are not tandemly arranged on daughter DNA strands. Biochemistry.

[158] Gershon L., Kupiec M. (2021). The Amazing Acrobat: Yeast’s Histone H3K56 Juggles Several Important Roles while Maintaining Perfect Balance. Genes.

[159] English C.M., Adkins M.W., Carson J.J., Churchill M.E.A., Tyler J.K. (2006). Structural Basis for the Histone Chaperone Activity of Asf1. Cell.

[160] Campos E.I., Fillingham J., Li G., Zheng H., Voigt P., Kuo W.-H.W., Seepany H., Gao Z., Day L.A., Greenblatt J.F. (2010). The program for processing newly synthesized histones H3.1 and H4. Nat. Struct. Mol. Biol..

[161] Schwabish M.A., Struhl K. (2006). Asf1 Mediates Histone Eviction and Deposition during Elongation by RNA Polymerase II. Mol. Cell.

[162] Driscoll R., Hudson A., Jackson S.P. (2007). Yeast Rtt109 Promotes Genome Stability by Acetylating Histone H3 on Lysine 56. Science.

[163] Franco A.A., Lam W.M., Burgers P.M., Kaufman P.D. (2005). Histone deposition protein Asf1 maintains DNA replisome integrity and interacts with replication factor C. Genes Dev..

[164] Su D., Hu Q., Li Q., Thompson J.R., Cui G., Fazly A., Davies B.A., Botuyan M.V., Zhang Z., Mer G. (2012). Structural basis for recognition of H3K56-acetylated histone H3–H4 by the chaperone Rtt106. Nature.

[165] Ben-Shahar T.R., Castillo A.G., Osborne M.J., Borden K.L.B., Kornblatt J., Verreault A. (2009). Two Fundamentally Distinct PCNA Interaction Peptides Contribute to Chromatin Assembly Factor 1 Function. Mol. Cell. Biol..

[166] Kondratick C.M., Litman J.M., Shaffer K.V., Washington M.T., Dieckman L.M. (2018). Crystal structures of PCNA mutant proteins defective in gene silencing suggest a novel interaction site on the front face of the PCNA ring. PLoS ONE.

[167] Li Q., Zhou H., Wurtele H., Davies B., Horazdovsky B., Verreault A., Zhang Z. (2008). Acetylation of Histone H3 Lysine 56 Regulates Replication-Coupled Nucleosome Assembly. Cell.

[168] Yang J., Zhang X., Feng J., Leng H., Li S., Xiao J., Liu S., Xu Z., Xu J., Li D. (2016). The Histone Chaperone FACT Contributes to DNA Replication-Coupled Nucleosome Assembly. Cell Rep..

[169] Gali V.K., Dickerson D., Katou Y., Fujiki K., Shirahige K., Owen-Hughes T., Kubota T., Donaldson A.D. (2018). Identification of Elg1 interaction partners and effects on post-replication chromatin re-formation. PLoS Genet..

[170] Hartman T., Stead K., Koshland D., Guacci V. (2000). Pds5p is an essential chromosomal protein required for both sister chromatid cohesion and condensation in *Saccharomyces cerevisiae*. J. Cell Biol..

[171] Kirchmaier A.L., Rine J. (2001). DNA Replication-Independent Silencing in *S. cerevisiae*. Science.

[172] Li Y.-C., Cheng T.-H., Gartenberg M.R. (2001). Establishment of Transcriptional Silencing in the Absence of DNA Replication. Science.

[173] Dodson A.E., Rine J. (2015). Heritable capture of heterochromatin dynamics in *Saccharomyces cerevisiae*. eLife.

[174] Janke R., King G.A., Kupiec M., Rine J. (2018). Pivotal roles of PCNA loading and unloading in heterochromatin function. Proc. Natl. Acad. Sci. USA.

[175] Peters J.-M., Nishiyama T. (2012). Sister Chromatid Cohesion. Cold Spring Harb. Perspect. Biol..

[176] Peters J.-M., Tedeschi A., Schmitz J. (2008). The cohesin complex and its roles in chromosome biology. Genes Dev..

[177] Nasmyth K. (2001). Disseminating the Genome: Joining, Resolving, and Separating Sister Chromatids during Mitosis and Meiosis. Annu. Rev. Genet..

[178] Nicklas R.B. (1988). The Forces that Move Chromosomes in Mitosis. Annu. Rev. Biophys. Biophys. Chem..

[179] Joglekar A.P., Hunt A.J. (2002). A Simple, Mechanistic Model for Directional Instability during Mitotic Chromosome Movements. Biophys. J..

[180] Hirano T. (2006). At the heart of the chromosome: SMC proteins in action. Nat. Rev. Mol. Cell Biol..

[181] Gruber S., Haering C.H., Nasmyth K. (2003). Chromosomal Cohesin Forms a Ring. Cell.

[182] Haering C.H., Löwe J., Hochwagen A., Nasmyth K. (2002). Molecular Architecture of SMC Proteins and the Yeast Cohesin Complex. Mol. Cell.

[183] Guacci V., Koshland D., Strunnikov A. (1997). A Direct Link between Sister Chromatid Cohesion and Chromosome Condensation Revealed through the Analysis of MCD1 in *S. cerevisiae*. Cell.

[184] Michaelis C., Ciosk R., Nasmyth K. (1997). Cohesins: Chromosomal Proteins that Prevent Premature Separation of Sister Chromatids. Cell.

[185] Haering C.H., Schoffnegger D., Nishino T., Helmhart W., Nasmyth K., Löwe J. (2004). Structure and Stability of Cohesin’s Smc1-Kleisin Interaction. Mol. Cell.

[186] Arumugam P., Gruber S., Tanaka K., Haering C.H., Mechtler K., Nasmyth K. (2003). ATP Hydrolysis Is Required for Cohesin’s Association with Chromosomes. Curr. Biol..

[187] Tanaka K., Hao Z., Kai M., Okayama H. (2001). Establishment and maintenance of sister chromatid cohesion in fission yeast by a unique mechanism. EMBO J..

[188] Chan K.-L., Gligoris T., Upcher W., Kato Y., Shirahige K., Nasmyth K., Beckouët F. (2013). Pds5 promotes and protects cohesin acetylation. Proc. Natl. Acad. Sci. USA.

[189] Sutani T., Kawaguchi T., Kanno R., Itoh T., Shirahige K. (2009). Budding Yeast Wpl1(Rad61)-Pds5 Complex Counteracts Sister Chromatid Cohesion-Establishing Reaction. Curr. Biol..

[190] Henrikus S.S., Costa A. (2021). Towards a Structural Mechanism for Sister Chromatid Cohesion Establishment at the Eukaryotic Replication Fork. Biology.

[191] Nasmyth K., Haering C.H. (2009). Cohesin: Its Roles and Mechanisms. Annu. Rev. Genet..

[192] Milutinovich M., Koshland D.E. (2003). Molecular biology: SMC Complexes—Wrapped Up in Controversy. Science.

[193] Eng T., Guacci V., Koshland D. (2015). Interallelic complementation provides functional evidence for cohesin–cohesin interactions on DNA. Mol. Biol. Cell.

[194] Srinivasan M., Scheinost J.C., Petela N.J., Gligoris T.G., Wissler M., Ogushi S., Collier J.E., Voulgaris M., Kurze A., Chan K.-L. (2018). The Cohesin Ring Uses Its Hinge to Organize DNA Using Non-topological as well as Topological Mechanisms. Cell.

[195] Xiang S., Koshland D. (2021). Cohesin architecture and clustering in vivo. eLife.

[196] Shi D., Zhao S., Zuo M.-Q., Zhang J., Hou W., Dong M.-Q., Cao Q., Lou H. (2020). The acetyltransferase Eco1 elicits cohesin dimerization during S phase. J. Biol. Chem..

[197] Uhlmann F., Lottspeich F., Nasmyth K. (1999). Sister-chromatid separation at anaphase onset is promoted by cleavage of the cohesin subunit Scc1. Nature.

[198] Uhlmann F., Wernic D., Poupart M.-A., Koonin E.V., Nasmyth K. (2000). Cleavage of Cohesin by the CD Clan Protease Separin Triggers Anaphase in Yeast. Cell.

[199] Ciosk R., Shirayama M., Shevchenko A., Tanaka T., Toth A., Shevchenko A., Nasmyth K. (2000). Cohesin’s Binding to Chromosomes Depends on a Separate Complex Consisting of Scc2 and Scc4 Proteins. Mol. Cell.

[200] Lengronne A., McIntyre J., Katou Y., Kanoh Y., Hopfner K.-P., Shirahige K., Uhlmann F. (2006). Establishment of Sister Chromatid Cohesion at the *S. cerevisiae* Replication Fork. Mol. Cell.

[201] Uhlmann F., Nasmyth K. (1998). Cohesion between sister chromatids must be established during DNA replication. Curr. Biol..

[202] Ivanov D., Schleiffer A., Eisenhaber F., Mechtler K., Haering C.H., Nasmyth K. (2002). Eco1 Is a Novel Acetyltransferase that Can Acetylate Proteins Involved in Cohesion. Curr. Biol..

[203] Hou F., Zou H. (2005). Two Human Orthologues of Eco1/Ctf7 Acetyltransferases Are Both Required for Proper Sister-Chromatid Cohesion. Mol. Biol. Cell.

[204] Zhang J., Shi X., Li Y., Kim B.-J., Jia J., Huang Z., Yang T., Fu X., Jung S.Y., Wang Y. (2008). Acetylation of Smc3 by Eco1 Is Required for S Phase Sister Chromatid Cohesion in Both Human and Yeast. Mol. Cell.

[205] Gandhi R., Gillespie P.J., Hirano T. (2006). Human Wapl Is a Cohesin-Binding Protein that Promotes Sister-Chromatid Resolution in Mitotic Prophase. Curr. Biol..

[206] Unal E., Heidinger-Pauli J.M., Kim W., Guacci V., Onn I., Gygi S.P., Koshland D.E. (2008). A Molecular Determinant for the Establishment of Sister Chromatid Cohesion. Science.

[207] Ben-Shahar T.R., Heeger S., Lehane C., East P., Flynn H., Skehel M., Uhlmann F. (2008). Eco1-Dependent Cohesin Acetylation During Establishment of Sister Chromatid Cohesion. Science.

[208] Rowland B.D., Roig M.B., Nishino T., Kurze A., Uluocak P., Mishra A., Beckouet F., Underwood P., Metson J., Imre R. (2009). Building Sister Chromatid Cohesion: Smc3 Acetylation Counteracts an Antiestablishment Activity. Mol. Cell.

[209] Guacci V., Chatterjee F., Robison B., Koshland D.E. (2019). Communication between distinct subunit interfaces of the cohesin complex promotes its topological entrapment of DNA. eLife.

[210] Moldovan G.-L., Pfander B., Jentsch S. (2006). PCNA Controls Establishment of Sister Chromatid Cohesion during S Phase. Mol. Cell.

[211] Bender D., Da Silva E.M.L., Chen J., Poss A., Gawey L., Rulon Z., Rankin S. (2020). Multivalent interaction of ESCO2 with the replication machinery is required for sister chromatid cohesion in vertebrates. Proc. Natl. Acad. Sci. USA.

[212] Feytout A., Vaur S., Genier S., Vazquez S., Javerzat J.-P. (2011). Psm3 Acetylation on Conserved Lysine Residues Is Dispensable for Viability in Fission Yeast but Contributes to Eso1-Mediated Sister Chromatid Cohesion by Antagonizing Wpl1. Mol. Cell. Biol..

[213] Borges V., Smith D.J., Whitehouse I., Uhlmann F. (2013). An Eco1-independent sister chromatid cohesion establishment pathway in *S. cerevisiae*. Chromosoma.

[214] Mayer M.L., Pot I., Chang M., Xu H., Aneliunas V., Kwok T., Newitt R., Aebersold R., Boone C., Brown G.W. (2004). Identification of Protein Complexes Required for Efficient Sister Chromatid Cohesion. Mol. Biol. Cell.

[215] Warren C.D., Eckley D.M., Lee M.S., Hanna J.S., Hughes A., Peyser B., Jie C., Irizarry R., Spencer F.A. (2004). S-Phase Checkpoint Genes Safeguard High-Fidelity Sister Chromatid Cohesion. Mol. Biol. Cell.

[216] Xu H., Boone C., Klein H.L. (2004). Mrc1 Is Required for Sister Chromatid Cohesion To Aid in Recombination Repair of Spontaneous Damage. Mol. Cell. Biol..

[217] Xu H., Boone C., Brown G.W. (2007). Genetic Dissection of Parallel Sister-Chromatid Cohesion Pathways. Genetics.

[218] Uzunova S.D., Zarkov A.S., Ivanova A.M., Stoynov S.S., Nedelcheva-Veleva M.N. (2014). The subunits of the S-phase checkpoint complex Mrc1/Tof1/Csm3: Dynamics and interdependence. Cell Div..

[219] Mohanty B.K., Bairwa N.K., Bastia D. (2006). The Tof1p-Csm3p protein complex counteracts the Rrm3p helicase to control replication termination of *Saccharomyces cerevisiae*. Proc. Natl. Acad. Sci. USA.

[220] Singh S., Shemesh K., Liefshitz B., Kupiec M. (2013). Genetic and physical interactions between the yeastELG1gene and orthologs of the Fanconi anemia pathway. Cell Cycle.

[221] Katheeja M.N., Das S.P., Laha S. (2021). The budding yeast protein Chl1p is required for delaying progression through G1/S phase after DNA damage. Cell Div..

[222] Porcella S.Y., Koussa N.C., Tang C.P., Kramer D.N., Srivastava P., Smith D.J. (2020). Separable, Ctf4-mediated recruitment of DNA Polymerase α for initiation of DNA synthesis at replication origins and lagging-strand priming during replication elongation. PLoS Genet..

[223] Yuan Z., Georgescu R., Santos R.D.L.A., Zhang D., Bai L., Yao N.Y., Zhao G., O’Donnell M.E., Li H. (2019). Ctf4 organizes sister replisomes and Pol α into a replication factory. eLife.

[224] Srinivasan M., Fumasoni M., Petela N.J., Murray A., Nasmyth K.A. (2020). Cohesion is established during DNA replication utilising chromosome associated cohesin rings as well as those loaded de novo onto nascent DNAs. eLife.

[225] Srinivasan M., Petela N.J., Scheinost J.C., Collier J., Voulgaris M., Roig M.B., Beckouet F., Hu B., Nasmyth K.A. (2019). Scc2 counteracts a Wapl-independent mechanism that releases cohesin from chromosomes during G1. eLife.

[226] Tanaka H., Kubota Y., Tsujimura T., Kumano M., Masai H., Takisawa H. (2009). Replisome progression complex links DNA replication to sister chromatid cohesion in Xenopusegg extracts. Genes Cells.

[227] Parnas O., Zipin-Roitman A., Mazor Y., Liefshitz B., Ben-Aroya S., Kupiec M. (2009). The Elg1 Clamp Loader Plays a Role in Sister Chromatid Cohesion. PLoS ONE.

[228] Maradeo M.E., Skibbens R.V. (2009). The Elg1-RFC Clamp-Loading Complex Performs a Role in Sister Chromatid Cohesion. PLoS ONE.

[229] Tong K., Skibbens R.V. (2015). Pds5 regulators segregate cohesion and condensation pathways in *Saccharomyces cerevisiae*. Proc. Natl. Acad. Sci. USA.

[230] Maradeo M.E., Skibbens R.V. (2010). Replication Factor C Complexes Play Unique Pro- and Anti-Establishment Roles in Sister Chromatid Cohesion. PLoS ONE.

